# Natural biological variation of white matter microstructure is accentuated in Huntington's disease

**DOI:** 10.1002/hbm.24191

**Published:** 2018-04-22

**Authors:** Sarah Gregory, Helen Crawford, Kiran Seunarine, Blair Leavitt, Alexandra Durr, Raymund A. C. Roos, Rachael I. Scahill, Sarah J. Tabrizi, Geraint Rees, Douglas Langbehn, Michael Orth

**Affiliations:** ^1^ Huntington's Disease Research Centre, UCL Institute of Neurology London United Kingdom; ^2^ Developmental Imaging and Biophysics Section UCL Institute of Child Health London WC1N 1EH United Kingdom; ^3^ Centre for Molecular Medicine and Therapeutics, Department of Medical Genetics University of British Columbia Vancouver BC V5Z 4H4 Canada; ^4^ APHP Department of Genetics Groupe Hospitalier Pitié‐Salpêtrière, and Institut du Cerveau et de la Moelle, INSERM U1127, CNRS UMR7225, Sorbonne Universités – UPMC Université Paris VI UMR_S1127 Paris France; ^5^ Department of Neurology Leiden University Medical Centre Leiden 2300RC The Netherlands; ^6^ Wellcome Trust Centre for Neuroimaging University College London London United Kingdom; ^7^ Departments of Psychiatry and Biostatistics University of Iowa Iowa City Iowa; ^8^ Department of Neurology Ulm University Hospital Ulm Germany

**Keywords:** Diffusion Tensor Imaging, Huntington's disease, natural variability

## Abstract

Huntington's disease (HD) is a monogenic neurodegenerative disorder caused by a CAG‐repeat expansion in the Huntingtin gene. Presence of this expansion signifies certainty of disease onset, but only partly explains age at which onset occurs. Genome‐wide association studies have shown that naturally occurring genetic variability influences HD pathogenesis and disease onset. Investigating the influence of biological traits in the normal population, such as variability in white matter properties, on HD pathogenesis could provide a complementary approach to understanding disease modification. We have previously shown that while white matter diffusivity patterns in the left sensorimotor network were similar in controls and HD gene‐carriers, they were more extreme in the HD group. We hypothesized that the influence of natural variation in diffusivity on effects of HD pathogenesis on white matter is not limited to the sensorimotor network but extends to cognitive, limbic, and visual networks. Using tractography, we investigated 32 bilateral pathways within HD‐related networks, including motor, cognitive, and limbic, and examined diffusivity metrics using principal components analysis. We identified three independent patterns of diffusivity common to controls and HD gene‐carriers that predicted HD status. The first pattern involved almost all tracts, the second was limited to sensorimotor tracts, and the third encompassed cognitive network tracts. Each diffusivity pattern was associated with network specific performance. The consistency in diffusivity patterns across both groups coupled with their association with disease status and task performance indicates that naturally‐occurring patterns of diffusivity can become accentuated in the presence of the HD gene mutation to influence clinical brain function.

## 1 INTRODUCTION

Huntington's disease (HD) is caused by a CAG‐repeat‐expansion in the Huntingtin gene (*HTT*). The expansion length is variable and explains much of the heterogeneity in the age at which mutation‐carriers receive a clinical diagnosis of manifest disease (Duyao et al., 1993). A large portion of variability in age at onset, however, remains unexplained by the gene‐mutation and is likely the result of other factors, both genetic and environmental (Wexler et al., 2004). Identifying these factors and their associated biological processes could provide additional targets for therapeutic interventions that may modify disease progression.

Approximately, half of the unexplained variability for age at onset may be heritable. It is therefore unsurprising that much focus has centered on the role of potential genetic modifiers that do not cause disease but nonetheless influence the effects of a disease‐causing mutated gene (GeM‐HD Consortium, 2015; Lee et al., 2012). Genome‐wide association analysis identified possible independent modifiers that may influence the age at clinical onset in HD (GeM‐HD Consortium, 2015). However, further work is required to understand the mechanisms of modifier genes in a comprehensive biological context. As a complementary approach, it would be valuable to first identify biological traits in the normal population that may influence HD pathogenesis and then investigate the underlying e.g., genetic basis. Similar to genomic variability the expression of biological traits, e.g., height or eye color, varies without causing disease. The expression of that biological trait could, however, influence the vulnerability to the effects of a mutated gene. TRACK‐HD and PREDICT‐HD, for example, reported changes in brain structure that were independent of *HTT* CAG repeat length and age (Paulsen et al., 2014; Tabrizi et al., 2013). The identification of biological traits could help reveal an interaction between the underlying biology and pathogenesis, or the timing of clinical manifestations of HD. Such traits, and their genetic bases, could propose a route to disease modification.

Macrostructural brain changes are a consistent phenotype in HD. In addition, considerable evidence points towards changes in white matter microstructure in HD cohorts. Diffusivity is an indirect marker of white matter tract organization. Increases of diffusivity across both the whole brain and in select white matter pathways, such as within the sensorimotor network, are suggestive of white matter degeneration in premanifest (preHD) and early manifest HD (Della Nave et al., 2010; Douaud et al., 2009; Dumas et al., 2012; Klöppel et al., 2008; Matsui et al., 2014; Novak et al., 2014; Odish et al., 2015; Poudel et al., 2014, 2015). In a recent study of the sensorimotor network, we observed a broad structural HD phenotype that encompassed gray and white matter volume, cortical thickness, and altered diffusivity in left white matter tracts in the sensorimotor network, which was associated with CAG repeat length (Orth et al., 2016). In addition, we identified an inverse relationship between levels of axial diffusivity (AD; water movement in the direction of the main tract) and radial diffusivity (RD; water movement perpendicular to the main tract) that predicted both motor performance and disease stage in HD and was independent of CAG repeat length. Interestingly, this same diffusivity pattern was also evident in controls, suggesting that it may reflect natural biological variation in white matter microstructure, which in the presence of the HD gene‐mutation independently contributes to HD pathogenesis.

Given the extant evidence of widespread white matter changes, we have investigated the potential role of natural biological variation on white matter microstructural alterations beyond those that we had described within the left sensorimotor network in HD. We tested to what extent the effects of natural variability on HD pathogenesis are specific only to sensorimotor white matter tracts most likely affected by HD pathology or whether there is a more widespread effect across the whole brain. We performed tractography analyses to investigate diffusivity in 32 tracts within networks likely to be affected as part of the HD phenotype: sensorimotor, cognitive, limbic (neuropsychiatric), and visual and subsequently employed principal components analysis (PCA) on diffusivity metrics extracted from these tracts to identify and parsimoniously describe diffusivity patterns in both controls and HD gene‐mutation carriers. First, we examined whether patterns differed between controls, preHD and earlyHD or if there was some consistency within the control and HD populations. Second, we aimed to distinguish diffusivity patterns linked to key contributors to HD pathology, e.g., CAG repeat length, from those that are independent but still influence HD manifestation. Third, we investigated the relative contribution of the three diffusivity measures i.e., RD, AD, and FA to each pattern of diffusivity or principal component. Finally, we examined the relationship between each PC and both cognitive and motor performance as indexed by the global cognitive composite and grip force, respectively. We predicted that tracts associated with networks more directly affected in HD, in particular those connecting striatal regions would likely show more disruption than cortico‐cortical tracts and that the main patterns of diffusivity would discriminate robustly between controls, preHD and earlyHD gene‐mutation carriers. We also predicted that patterns of natural variation would be associated with HD‐related biological variables, including volume changes and CAG‐repeat length extending to structural connections in other brain networks and therefore representing a wider structural HD phenotype.

## 2 METHODS AND MATERIALS

### 2.1 Participants

We analyzed brain images from the TrackOn‐HD study. Participants were recruited into the TrackOn‐HD study at four study sites (London, Paris, Leiden, Vancouver) as previously described (Kloppel et al., 2015; Orth et al., 2016). For the present analyses, we used data of those participants who had complete Diffusion Tensor Imaging (DTI) data. Sixty‐one individuals (*F* = 44%; mean age ± SD: 43.1 ± 9.1) carried the *HTT* gene with a CAG trinucleotide repeat expansion mutation of ≥ 39 but did not have a clinical diagnosis of HD. Thirteen participants (*F* = 62%, mean age ± SD: 43.1 ± 3.3) had early motor manifest HD (UHDRS diagnostic confidence level of 3 or 4), and 79 were controls (*F* = 61%, mean age ± SD: 49.1 ± 9.7). A global cognitive composite score was derived from nine cognitive tasks that were completed in testing sessions separate to the MRI procedures: Stroop Word Reading test, Symbol Digit Modality Test, Paced Tapping, Circle Tracing (two conditions), Map Search test, Cancelation task, the Spot the Change visual working memory task, Mental Rotation task (Jones et al., 2014). The United Huntington's Disease Rating Scale (UHDRS) motor examination was administered to all participants to derive the UHDRS total motor score. Grip force as a sensorimotor test was selected as a further marker of motor performance; for details see (Kloppel et al., 2015; Orth et al., 2016). All participants were right‐handed. Exclusion criteria included age below 18 or above 65 (unless previously in Track‐HD study), major psychiatric, neurological or medical disorder or a history of severe head injury (Kloppel et al., 2015). The study was approved by the local ethics committees, and all participants gave written informed consent according to the Declaration of Helsinki.

### 2.2 MRI data acquisition and analysis

Standardisation of data acquisition across sites was performed based on previous suggestions (Glover et al., 2012; Kloppel et al., 2015). 3T‐MRI data were acquired on two different scanner systems (Philips Achieva at Leiden and Vancouver and Siemens TIM Trio at London and Paris). Diffusion‐weighted images were collected with 42 unique gradient directions (*b* = 1,000 s/mm^2^) with eight images with no diffusion weighting (*b* = 0 s/mm^2^) (Siemens) and one image with no diffusion weighting (*b* = 0 s/mm^2^) (Philips). Acquisition parameters were TE = 88 ms, TR = 13 s and voxel size 2 × 2 × 2 mm (Siemens); TE = 56ms and TR = 11s and voxel size 1.96 × 1.96 × 2.75 mm (Philips). The diffusion data were preprocessed using standard FSL pipelines (Smith et al., 2004).

The T1 scan was segmented into gray and white matter using the VBM8 toolbox (http://dbm.neuro.uni-jena.de/vbm/) and combined to create an improved anatomical scan for DTI data registration. Prior to analysis, we screened each DTI dataset for artifacts, signal drop‐out and motion. Data were then corrected for distortions caused by eddy currents and motion using eddy_correct in FSL, and vector gradient information updated accordingly. The no‐gradient (B0) image (an averaged B0 image was used for the Siemens data) was then skull‐stripped using the Brain Extraction Tool (BET) and manually corrected. For registration purposes, we also applied BET to the structural T1 image. To improve the quality of the brain mask, we combined and dilated a thresholded segmented image with an eroded brain‐extracted T1 mask, which was then applied to the original brain‐extracted T1 image. We then linearly registered the resultant T1 image to the B0 image using FLIRT (Jenkinson & Smith, 2001) with standard parameters. Diffusion tensors were fit to the corrected data using dtifit and fractional anisotropy (FA) axial diffusivity (AD) and radial diffusivity (RD) values derived. We modeled within‐voxel crossing fibers using a Bayesian probabilistic method implemented in Bedpostx (Behrens et al., 2003).

Probabilistic tractography was performed for a series of tracts using probtrackx (Behrens, Berg, Jbabdi, Rushworth, & Woolrich, 2007); more information can be found at Table 1. For the sensorimotor network, this included tracts in the right hemisphere connecting the primary motor cortex (M1) and the motor thalamus; the premotor cortex (PMC) and the motor thalamus; and the primary somatosensory cortex (S1) and the somatosensory thalamus (for completeness we also included the equivalent left‐sided tracts from our previous study in the final analyses). This was then repeated replacing the thalamic region with the putamen in both hemispheres. Tracking was then performed on bilateral tracts within the cognitive network connecting the dorsolateral prefrontal cortex (DLPFC) and the DLPFC thalamic parcellation and caudate, respectively; and the posterior parietal cortex (PPC) and the parietal thalamus. We also performed tractography on tracts within “limbic circuits,” the middle and posterior cingulum and the uncinate fasciculus (UF) bilaterally. Finally, we performed fiber‐tracking between the primary visual cortex (V1) and the visual thalamus, the inferior fronto‐occipital fasciculus (IFOF), the inferior longitudinal fasciculus (ILF) and the middle and posterior corpus callosum (see Supporting Information Table S1 for further details). Masks were created using the Anatomy Toolbox, the WFU PickAtlas (Maldjian, Laurienti, Kraft, & Burdette, 2003) or FSL MNI template and the JHU White Matter Labels atlas (Wakana et al., 2007). All masks were defined in standard MNI space and warped into native space for each participant. Exclusion masks were used to exclude any streamlines that may track via the contralateral hemisphere or outside of the anatomically‐defined tract. A white matter termination mask was also used to ensure tracts stopped at the gray/white matter interface and did not extend beyond the white matter into the gray matter, CSF or dura. The resulting tracts were then warped into diffusion space using FLIRT. All tracts were visually inspected following probtrackx analyses and warping into diffusion space. Due to poor tracking in a high number of participants, the following tracts were excluded from the final analyses: the PPC and the parietal thalamus, the lateral orbitofrontal cortex to the caudate, the anterior cingulum and the anterior corpus callosum. Mean fractional anisotropy (FA), axial diffusivity (AD), and radial diffusivity (RD) values were extracted from the mask of each tract for each participant weighted by the contribution of each voxel by the number of streamlines that pass through it (as opposed to assuming an equal contribution from each voxel).

**Table 1:** Tracts included in principal component analysis

Network	Tract
Sensorimotor network	Primary Motor Cortex (M1) – Putamen
	Primary Motor Cortex (M1) – Motor Thalamus
	Premotor Cortex – Putamen
	Premotor Cortex – Motor Thalamus
	Primary Somatosensory Cortex (S1) – Putamen
	Primary Somatosensory Cortex (S1) – Somatosensory thalamus
Visual network	Inferior Fronto‐Occipital Fasciculus
	Primary Visual Cortex (V1) – Visual Thalamus
Limbic network	Uncinate Fasciculus
	Mid Cingulum
	Posterior Cingulum
Cognitive network	Dorsolateral Prefrontal Cortex – Caudate
	Dorsolateral Prefrontal Cortex –Prefrontal Thalamus
	Posterior Parietal Cortex – Parietal Thalamus
“Non‐HD”	Inferior Lateral Fasciculus
Interhemispheric tracts	Mid corpus callosum
	Posterior corpus callosum

Tractography was performed for all listed tracts. Diffusivity measures were extracted from each tract and included in our PCA analysis.

### 2.3 Statistical analysis

We performed PCA using centered, standardized (standard deviation = 1) versions of the tract‐specific DTI measures. PCA is a method to identify patterns of correlation among multiple measures and is based on the correlations among those measures. To the extent that the first few principal components account for much of the combined variance in a set of variables, those components serve as a useful approximation of the overall correlation patterns. Principal components are described in terms of their correlations with their constituent variables, and the pattern represented by a component can be understood in terms of these correlations. Furthermore, participants’ original data can be transformed to scores on each principal component. We can then analyze a few component scores rather than a large number of original measures, for example, to examine group differences. This reduces multiple comparisons and ideally contributes to insight regarding systematic patterns that may be related to underlying biology and pathology.

Initially, we observed that separate PCA of the controls and of the combined preHD and earlyHD group yielded very similar results (see Supporting Information Table S2 for further details). Subsequently, the final PCA was performed on all three groups combined. The data were first screened for univariate outliers with the only cases identified from tracts subsequently removed from the analyses due to general QC issues as described above. As a check of stability and potential multivariate outlier influence, PCA was also performed using Hubert's robust PCA (Hubert, Rousseeuw, & Vanden Branden, 2005) as implemented in the R packaged *rrcov* (Todorov & Filzmoser, 2009). These results (not shown) were in excellent agreement with conventional PCA and influence plots suggested no substantial outliers. Differences in PC scores among groups were assessed via ANOVA. Group differences and their statistical significance were calculated from the ANOVA‐based means and standard errors. Statistical significance was defined as *p* < .05, after Tukey‐Kramer adjustment for multiple group comparisons. Predictive models for PC scores were all estimated by linear regression. We used logistic regression for multivariate prediction of earlyHD versus preHD clinical status. We used Pearson correlation (equivalent to linear regression), with and without control for age and CAG, to assess relationships between DTI scores and cognitive or motor measures. With the exception noted above, all analyses were performed with SAS/STAT 14.1 (SAS Institute Inc., Cary, NC).

## 3 RESULTS

**Figure 1:**
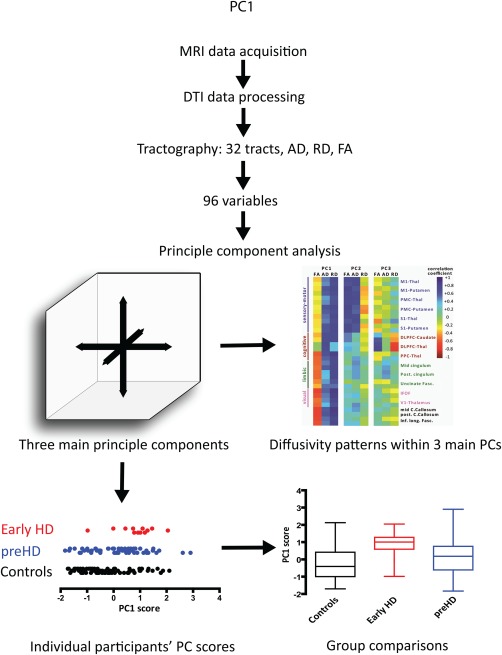
Study workflow. After processing data from the DTI sequence, tractography was employed to discern 15 tracts bilaterally, the mid corpus callosum and posterior corpus callosum so that, altogether, we derived 32 tracts. From each tract we extracted information about axial (AD) and radial diffusivity (RD) as well as fractional anisotropy (FA). Hence, we included 96 diffusivity measures in our PCA. The first 3 principle components, each of which contains information on all 3 diffusivity measures (AD, RD, FA) explained 56% of the variability in the data. The heatmap illustrates the relative contribution of each tract's diffusivity measures to the respective PC to show the diffusivity patterns for each PC across all tracts. For the three main PCs, we then calculated a principle component score for each participant followed by group comparisons of PCs [Color figure can be viewed at http://wileyonlinelibrary.com]

We investigated diffusivity in 32 tracts within networks likely to be affected as part of the HD phenotype: sensorimotor, cognitive, limbic (neuropsychiatric), and visual (Table 1; Figure 1). We employed PCA in all participants combined to identify diffusivity patterns and addressed three main questions. First, we examined the diffusivity patterns across the 32 white matter tracts and asked whether such patterns differed between controls, preHD and earlyHD. Second, we aimed to distinguish diffusivity patterns linked to key contributors to HD pathology, e.g., CAG repeat length, from those that are independent but still influence HD manifestation. Third, we investigated the relative contribution of the three diffusivity measures i.e., RD, AD, and FA to each pattern of diffusivity or principal component. Finally, we examined the relationship between each PC and both cognitive and motor performance as indexed by the global cognitive composite and grip force, respectively.

### 3.1 Diffusivity patterns (PCA) across both controls and HD gene‐carriers

**Figure 2:**
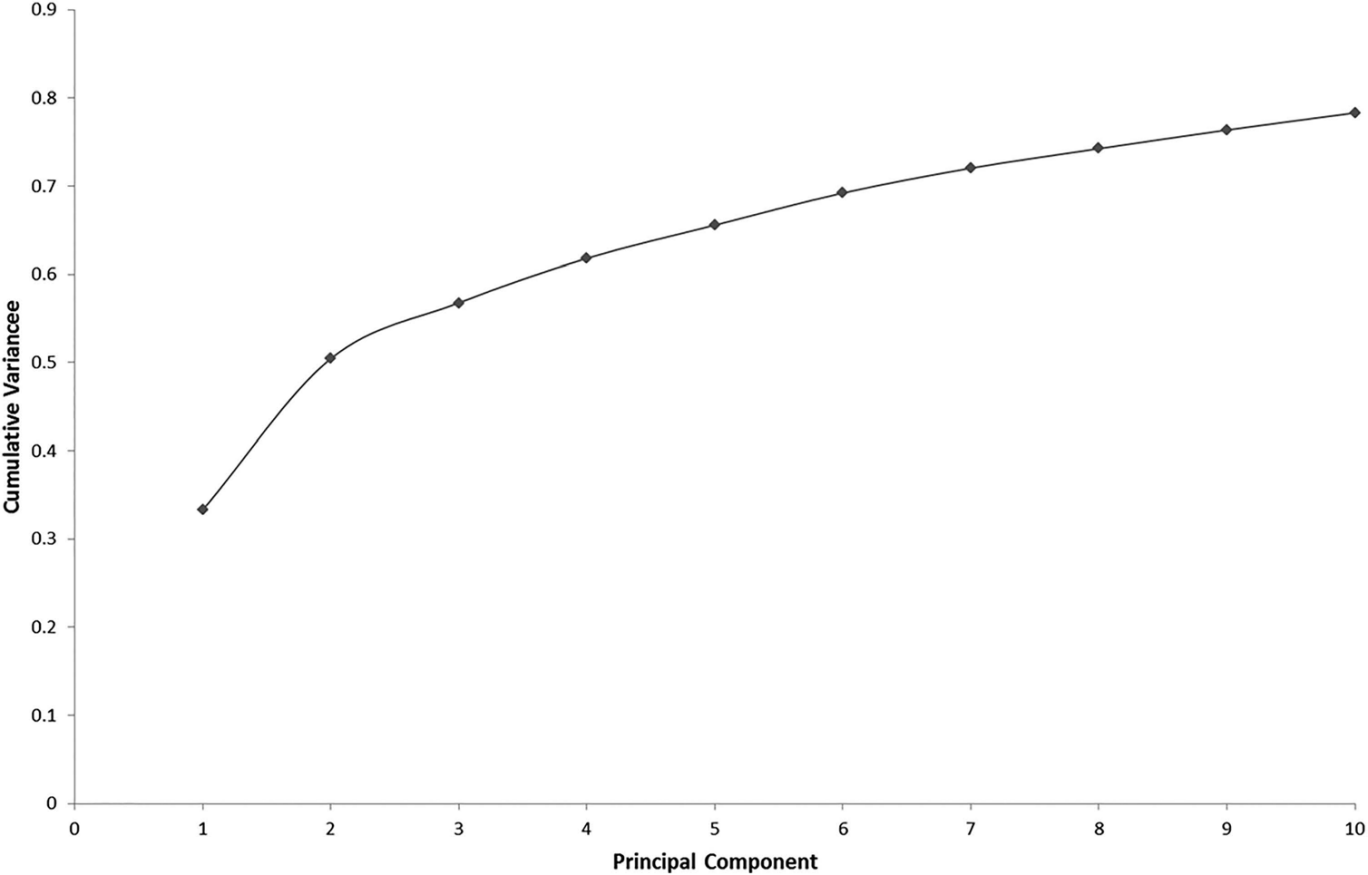
Principal component analysis. Scree plot showing variance explained by the first 10 principal components

We first examined diffusivity patterns, i.e., PC (principal components) across all 32 tracts of the combined control and HD gene‐carrier groups. PCA revealed that the first three principal components explained 56.8% of the overall variance in diffusivity (Figure 2). Thus, the three corresponding patterns of diffusion metric correlations describe a substantial portion of the overall diffusion metric variability within the underlying white‐matter regions. As noted in the Methods, the first three PCs of the joint data closely resembled the corresponding PCs when the controls and HD groups were analyzed separately. The similarity with the PCA of controls alone suggests that the overall variability in the data forms patterns similar to the patterns of correlated natural variability within the healthy white matter of the controls. Nonetheless, within the joint analysis there were notable differences in the mean PC scores. On the first and third PCs, the early HD group had significantly different mean scores compared to the preHD and control groups (see below).

**Figure 3:**
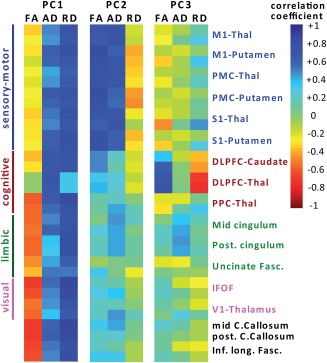
Principal component analysis of diffusivity. Heat map of correlation coefficients for each measure of diffusivity with dimensions derived from PCA performed in controls and HD participants. For each tract, there are two rows within the heat map; the top represents the left hemisphere, the bottom the right hemisphere. Abbreviations: DLPFC, dorsolateral prefrontal cortex; IFOF, inferior fronto‐occipital fasciculus; M1, primary motor cortex; PMC, premotor cortex; PPC, posterior parietal cortex; S1, primary sensorimotor cortex; V1, primary visual cortex [Color figure can be viewed at http://wileyonlinelibrary.com]

For PC1, there was a pattern across all tracts which demonstrated an inverse relationship between FA and the two diffusivity measures, AD and RD, i.e., the lower the FA, the higher the AD and RD and vice‐versa (Figure 3; Table 2). This was particularly the case in connections from the PPC to the thalamus, limbic tracts (the mid and posterior cingulum and the uncinate fasciculus), visual tracts (IFOF, V1 to thalamus), the corpus callosum and the ILF. Additionally, AD and RD were correlated with PC1 in all sensorimotor tracts (bilateral M1, PMC, and S1 to putamen and thalamus) and cognitive tracts (DLPFC to caudate and thalamus, respectively). However, FA reductions in the sensorimotor and cognitive tracts were not as high as those in the limbic and visual tracts, the corpus callosum or the ILF (Figure 3). PC1 scores distinguished clinical groups. Scores were higher in early HD compared to controls (0.61 versus −0.24, *p* = .0002) and to the preHD group (0.61 versus 0.05, *p* = .0395) while the scores of controls and preHD were similar (–0.24 versus 0.04, *p* = .238). Thus, the pattern of diffusivity in PC1 was significantly more pronounced in earlyHD (Figure 3).

**Table 2:** PCA results [Color table can be viewed at http://wileyonlinelibrary.com]

		PC1			PC2			PC3		
		FA	AD	RD	FA	AD	RD	FA	AD	RD
M1‐Motor Thalamus	L		0.4944	0.71558	0.67955	0.70197				
	R		0.43883	0.67178	0.64759	0.71228				
M1‐Putamen	L		0.59931	0.77047	0.78749	0.61072				
	R		0.5695	0.66923	0.77874	0.66393				
PMC‐Motor Thalamus	L		0.43661	0.61912	0.73059	0.71341				
	R		0.45098	0.64774	0.68891	0.68221				
PMC‐Putamen	L		0.63268	0.74575	0.79596	0.58385	−0.43091			
	R		0.59244	0.67459	0.77046	0.55995	−0.46135			
S1‐Somatosensory Thalamus	L		0.51495	0.72899	0.70752	0.68283				
	R		0.46951	0.66197	0.61133	0.70453		−0.43991		0.46866
S1‐Putamen	L		0.59981	0.77641	0.77865	0.61116				
	R		0.62317	0.76785	0.75927	0.60292				
DLPFC‐Prefrontal Thalamus	L		0.69285					0.65991		−0.67064
	R		0.6591					0.74847		−0.74165
DLPFC‐Caudate	L		0.68251	0.68763	0.48431					
	R		0.66408	0.69064	0.47344			0.59726		−0.42632
PPC ‐ Parietal Thalamus	L	−0.57012	0.6499	0.83471						
	R	−0.54872	0.55881	0.75936		0.41155				
Uncinate Fasciculus	L	−0.4484	0.43608	0.61483						
	R		0.50387	0.50397						
Mid Cingulum	L	−0.54713	0.47198	0.71255		0.4385				
	R	−0.50473	0.56258	0.77297		0.40841			0.4084	
Posterior Cingulum	L	−0.5396		0.76733		0.42102			0.44304	
	R	−0.53816		0.76758		0.4282			0.41278	
V1 ‐ Visual Thalamus	L	−0.52886	0.44216	0.74869						
	R	−0.53247		0.65093		0.44095				
IFOF	L	−0.65731	0.5482	0.84464						
	R	−0.60571	0.49602	0.81886						
ILF	L	−0.63733	0.50695	0.77461						
	R	−0.61165	0.47702	0.79844						
Mid Corpus Callosum		−0.67649	0.57568	0.84759		0.41806				
Posterior Corpus Callosum		−0.6755	0.61222	0.82682						

Correlation co‐efficients for each PC showing the contribution of each measure of diffusivity to each PC for all tracts**;** for visualization purposes, these have only been shown for co‐efficients that are smaller than or greater than 0.4. Tracts are color‐coded according to the network to which they putatively belong. Blue: Sensorimotor Network tracts; Green: Cognitive network tracts; Pink: Limbic network tracts; Orange: Visual network tracts; Purple: Control (non‐HD phenotype); Brown: Interhemispheric tracts. AD: axial diffusivity; DLPFC: dorsolateral prefrontal cortex; FA: fractional anisotropy; IFOF: inferior fronto‐occipital Fasciculus; ILF: inferior lateral Fasciculus; M1: primary motor cortex; PMC: premotor cortex; PPC: posterior parietal cortex; RD: radial diffusivity; S1: primary somatosensory cortex; V1: primary visual cortex.

PC2 revealed a diffusivity pattern of increased FA and AD focused mainly on sensorimotor tracts (Figure 3; Table 2), while RD showed little association. PC2 scores were significantly higher in earlyHD than controls (0.48 versus −0.19, *p* = .006) and also higher than those in preHD, although the difference was not statistically significant (0.48 versus −0.04, *p* = .148). Again, there was no significant difference in scores between controls and preHD (–0.19 versus 0.04, *p* = .395). Thus, the pattern of diffusivity in PC2 was significantly more pronounced in earlyHD.

Finally, PC3 revealed a diffusivity pattern of increased FA and reduced RD in bilateral connections between the DLPFC and caudate and thalamus, respectively (Table 2), while increased AD correlated with PC3 in the right mid and bilateral posterior cingulum. PC3 scores were lower in earlyHD than both controls (–0.06 versus 0.03 *p* = .0016) and preHD (–0.06 versus 0.34, *p* = .0001) and there was no significant difference between controls and preHD (0.03 versus 0.34, *p* = .08). Thus the pattern of diffusivity in PC3 was lesser in earlyHD.

### 3.2 Associations between diffusivity patterns and HD pathology

As age and CAG‐repeat length interactions influence HD pathogenesis, we used linear regression to test whether age and CAG‐repeat length were associated with our diffusivity patterns. In the combined preHD and early HD group, the diffusivity pattern evident in PC1 was strongly predicted by both age (0.083 (0.02), *t* = 4.16, *p* = .0001) and CAG‐repeat length (0.291 (0.08), *t* = 3.62, *p* = .0005) but not an age‐by‐CAG interaction. There was a suggestion of a relationship between PC2 and CAG‐repeat length (0.094 (0.06), *t* = 1.71, *p* = .092), but no relationship with age; PC3 was predicted by age (0.039 (0.01), *t* = 2.76, *p* = .007) but not CAG‐repeat length. For controls, PC3 was significantly predicted by age (–0.027 (0.01), *t* = 2.52, *p* = .014), and there was a suggestion of age related difference in PC1 scores (0.02 (0.01), *t* = 1.86, *p* = .07). PC2 scores were not predicted by age.

Brain volume loss is a hallmark of HD progression and so we assessed whether diffusivity patterns distinguished preHD from earlyHD independently of whole brain, gray matter, white matter, putamen, caudate, and ventricular volumes. A preliminary model selection demonstrated that only the caudate and lateral ventricle volumes acted as joint predictors of HD status. Adding PC scores to that model, the diffusivity pattern of PC3 was also a significant predictor of disease status (log odds ratio = 1.078 per SD (0.410), *X* ^2^ = 6.90, *p* = .009), but PC1 (log odds ratio = 0.166 per SD (0.38) *X* ^2^ = 0.192, *p* = .66) and PC2 (log odds ratio = 0.448 per SD (0.4) *X* ^2^ = 1.13, *p* = .29) were not.

### 3.3 Contribution of diffusivity measures to principal component patterns

**Figure 4:**
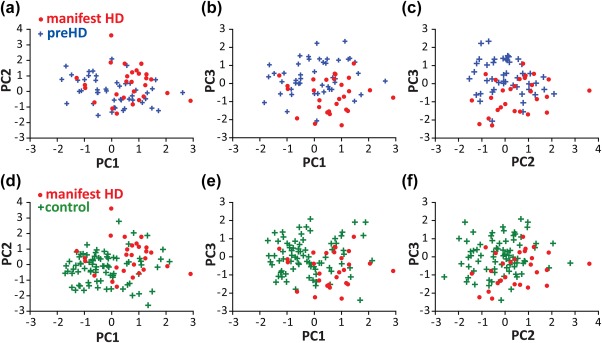
Relationships between PC scores. Scatterplots showing the relationships between individual PC scores for manifest HD (red dots) and preHD (blue crosses) (A‐C) and manifest HD and controls (green crosses) (D‐F). Manifest HD participants tend to have higher PC1 and PC2 scores, and lower PC3 scores, than preHD or control participants [Color figure can be viewed at http://wileyonlinelibrary.com]

PC coefficients represent correlations between diffusivity measures and their contributions to each pattern of diffusivity. We investigated these associations in a subset of representative tracts for each PC, plotting diffusivity metric values against PC scores with individual data points color‐coded for group membership. As discussed earlier, there was considerable overlap in distribution across the groups (Figure 4). For PC1, all three groups displayed correlations between PC1 scores and RD and AD in sensorimotor (bilateral PMC and both thalamus and putamen, respectively) and visual network (bilateral IFOF) tracts plus bilateral ILF (Figure 3; Table 2), suggesting that both AD and RD contributed strongly to PC1 regardless of the type of tract (Figure 3). Diffusivity values and mean scores were highest in earlyHD (Figure 4). For PC2 all three groups displayed a strong positive correlation between PC2 scores and both FA and AD in bilateral PMC and both thalamic and putaminal tracts with a weak negative correlation with RD. The differentiation between early HD and the other groups is more difficult to appreciate. Finally, for PC3, all three groups displayed a moderate negative correlation between RD and PC3 with the earlyHD group showing higher levels of RD and lower PC3 scores. AD also correlated moderately positively with PC3 scores in the bilateral mid and posterior cingulum and here, the earlyHD group had lower levels of FA and AD and lower PC scores compared to the control and preHD groups (Figure 4).

### 3.4 Relationship between diffusivity patterns and behavior

Global cognitive composite scores and grip force index (as a marker of motor performance) were correlated with overall PC scores for both controls and a combined HD group (Table 3). The diffusivity pattern of PC1 was negatively correlated with global cognition and positively correlated with motor performance for the combined HD group and to a lesser extent the control group, suggesting that widespread increased diffusivity was associated with diminished cognitive and motor performance. Associations for cognition were only slightly attenuated when controlling for the age‐CAG interaction and thus cannot be explained by the common causal influence of the HD gene. The sensorimotor‐based diffusivity pattern for PC2 was positively correlated with motor performance only in both groups with little effect of age or CAG. Finally, the cognition‐based diffusivity pattern for PC3, was not associated with either cognition or motor performance for either controls or HD.

**Table 3:** Correlations between PC scores and performance

	HD		Controls	
Global Cognitive Score		Adjusted		Adjusted
PC1 Score (74)	−0.449	−0.396	−0.199	−0.121
*p* val	<.0001	0.0006	0.0787	0.293
PC2 Score (74)	−0.133	−0.130	0.106	0.076
*p* val	0.2593	0.2785	0.354	0.5063
PC3 Score (74)	0.093	0.057	0.227	0.119
*p* val	0.4315	0.6396	0.0444	0.3008
RD: Left DLPFC‐Caud(90)	−0.304	−0.230	−0.311	−0.141
*p* val	0.0035	0.0321	0.0036	0.2012
RD: Right DLPFC‐Caud(90)	−0.284	−0.198	−0.271	−0.111
*p* val	0.0066	0.066	0.0115	0.3135
RD: Left DLPFC‐Thal(90)	−0.165	−0.103	−0.282	−0.210
*p* val	0.1204	0.3422	0.009	0.0547
RD: Right DLPFC‐Thal(90)	−0.101	−0.039	−0.218	−0.126
*p* val	0.3412	0.7196	0.0439	0.254

	HD	Controls		
Grip Force		Adjusted		Adjusted
PC1 Score (74)	0.361	0.349	0.196	0.203
*p* val	0.0016	0.0028	0.0839	0.0742
PC2 Score (74)	0.253	0.232	0.219	0.218
*p* val	0.0297	0.0517	0.053	0.0551
PC3 Score (74)	−0.015	0.006	0.117	0.117
*p* val	0.8993	0.9578	0.306	0.3077
				
AD: Left PMC‐Thal(74)	0.420	0.397	0.168	0.171
*p* val	0.0002	0.0006	0.1377	0.1334
AD: Right PMC‐Thal(90)	0.318	0.265	0.267	0.269
*p* val	0.0022	0.0257	0.0131	0.0127
AD: Left PMC‐Put(90)	0.407	0.464	0.141	0.146
*p* val	<.0001	<.0001	0.1946	0.1825
AD: Right PMC‐Put(90)	0.297	0.237	0.204	0.208
*p* val	0.0044	0.0464	0.0594	0.0557
RD: Left PMC‐Thal(74)	0.138	0.112	0.064	0.064
*p* val	0.2415	0.3507	0.5757	0.5791
RD: Right PMC‐Thal(90)	0.060	0.105	0.025	0.031
*p* val	0.5734	0.3857	0.8164	0.7758
RD: Left PMC‐Put(90)	0.126	0.147	0.108	0.128
*p* val	0.2355	0.2216	0.3223	0.2448
RD: Right PMC‐Put(90)	0.094	0.122	0.009	0.023
*p* val	0.378	0.3096	0.9324	0.8334

Pearson correlations between behavioral measures and PC Scores and individual diffusivity measures in specified tracts for HD and Control groups. Correlations are presented unadjusted and adjusted for each group; for HD the correlations are adjusted for Age, CAG, Age*CAG, for Controls the correlations are adjusted for Age. AD, axial diffusivity; DLPFC, Dorsolateral Prefrontal Cortex; PMC, premotor cortex; Put, Putamen; RD, radial diffusivity.

Looking at specific tracts, there were correlations between increased AD in PMC tracts, and reduced motor performance bilaterally for HD and in the right hemisphere for controls, that were little diminished when controlling for age and CAG repeat length. RD in the DLPFC—caudate tract was positively correlated with the global cognition in both groups. This was attenuated when controlling for age and CAG and was large enough in controls to suggest that changes may be due to ageing. However, many of the attenuated correlations remained significant in HD. There was also a similar correlation for the DLPFC–thalamic tract for controls but not HD.

## 4 DISCUSSION

In this study, we examined the relationships between AD, RD, and FA within white matter tracts in HD gene expansion mutation‐carriers and healthy controls and identified three independent patterns of diffusivity relationships common to both. Absolute diffusivity levels varied between individuals, while the relationship between AD, RD, and FA was maintained potentially representing natural biological variation of white matter microstructure. All three patterns were more pronounced in HD compared to controls; with two closely related to biological factors associated with the HD gene‐mutation. The first described a widespread pattern of increased AD, RD and reduced FA, while the second was restricted to increased AD and FA in sensorimotor network tracts. In the case of the HD group, these patterns were to some extent driven by CAG repeat length, the primary predictor of HD onset, indicating that HD pathogenesis may accentuate pre‐existing diffusivity patterns that are independent of the HD gene mutation and also present in controls. Further, both patterns were linked to widespread HD‐related brain volume loss reflecting key elements of the structural phenotype of HD. The third pattern, consisting mainly of RD changes in tracts associated with cognitive function, however, while associated with age was independent of both HD‐mediated volume loss and CAG repeat length, but nonetheless, a predictor of HD diagnostic status independently contributing to HD clinical manifestations.

In this study, we examined diffusivity across 32 white matter tracts from a number of functional networks and identified three independent patterns of AD, RD and FA relationships common to controls, preHD and earlyHD gene‐carriers. Having previously shown that in left‐sided sensorimotor tracts diffusivity naturally varies for both controls and HD gene‐carriers independently i.e., absolute diffusivity levels differs between individuals but the relationship between AD and RD is maintained (Orth et al., 2016), the current findings extend this concept of variability within naturally‐occurring patterns of diffusivity in HD to include pathways within cognitive, limbic and visual networks. This subsequently supports the idea that HD pathogenesis does not necessarily abolish pre‐existing patterns of diffusivity, or even generate new ones, but instead modifies existing patterns throughout the brain.

The patterns of diffusivity that we previously identified in the sensorimotor network, not only reflect natural variability but are also influenced by HD pathology and could be clinically relevant since they were associated with manifestations of unequivocal motor signs of HD (Orth et al., 2016). In the current study, therefore, we explored whether the exacerbation of such diffusivity patterns could potentially be considered a factor in HD pathology. Despite the commonalities between diffusivity patterns across the three groups, it is important to note that the distribution of the data along the PC axes, which describe the relationship between data, i.e., the pattern, differed between the earlyHD and control groups. Thus, differences in PC scores between groups indicate their placement at opposite ends of the axis. Indeed all three diffusivity patterns were accentuated in earlyHD, while remaining broadly similar for controls and preHD. For example, for PC1, where we saw a widespread pattern of increased AD and RD and to a lesser extent decreased FA across all 32 white matter tracts, the earlyHD group displayed higher scores mainly due to higher levels of RD compared to both controls and preHD. Similarly, the earlyHD group showed higher levels of both RD in cognitive tracts (PC3) and AD in bilateral sensorimotor tracts (PC2). In all cases, differences were only found in earlyHD, and there were no detectable differences in the preHD group. The evidence of both widespread and localized changes in diffusivity over and above those patterns seen in controls and preHD suggests that in the manifest stages of HD there is ongoing disorganization in white matter pathways which underscores a series of functions including motor and cognitive – both key elements of the phenotype. It is interesting to note that while there were no differences between preHD and controls, the direction of diffusivity changes in these naturally‐occurring patterns in the preHD was comparable with that seen in earlyHD. It is likely that the white matter changes in the earlyHD group begin many years prior to diagnosis but are not necessarily detectable in comparisons with non‐CAG expanded individuals. There is robust evidence to suggest that macrostructural white matter changes in the cortex occur prior to those in the gray matter with similar white matter microstructural changes (Paulsen et al., 2010; Tabrizi et al., 2012; Wu et al., 2017).

We also examined the extent to which the diffusivity patterns predict HD disease status independent of genetic and structural markers of HD progression. As such, despite significantly discriminating between preHD and earlyHD, both patterns of diffusivity identified in PC1 and PC2 were closely associated with CAG repeat length and whole brain, gray matter, white matter, putamen, and caudate volume loss and therefore, not independently associated with HD clinical group classifications. Here, it would appear that widespread increased radial diffusivity (as seen in PC1) and increased axial diffusivity in the sensorimotor network (as seen in PC2) were both closely related to the overall effects of HD pathogenesis, ultimately contributing to the structural phenotype of HD.

The third DLPFC‐based diffusivity pattern associated with cognitive function did, however, remain associated with disease status even when controlling for volumetric markers of HD pathology and CAG repeat length. Changes in this pattern may be due to factors that do not cause HD and the diffusivity properties represented may instead independently influence the effects of the HD mutation. As this particular pattern of diffusivity was associated with age for both controls and HD gene‐carriers, changes here may be related to normal aging. In other words, factors associated with aging may influence the diffusivity properties of frontal lobe white matter tracts involved in networks sub‐serving cognition. In healthy individuals, frontal lobe gray and white matter structure and connectivity have been associated with age‐related cognitive performance loss (Kievit et al., 2014; Serbruyns et al., 2016; Zhao et al., 2015) that may be associated with reduced myelination of the frontal lobe. Given the known benefits of cognitive training on myelination in healthy individuals (Caeyenberghs et al., 2016; Takeuchi et al., 2010), similar therapeutic interventions in HD gene‐carriers may counteract the effects of age and thus positively influence HD pathogenesis before disease onset, akin to the disease‐modifying effects of environmental enrichment in HD animal models (Dersi et al., 2016; Nithianantharajah and Hannan, 2006; Xu et al., 2016). Although this pattern of diffusivity correlated with global cognition in controls only, increased RD in the DLPFC‐caudate tract specifically correlated with cognitive performance in both controls and HD. Furthermore, this effect was reduced once correcting for age and CAG suggesting that while influenced by natural variability, the presence of the HD gene mutation has an independent effect that requires further investigation.

HD may not, however, affect all aspects of white matter microstructure in equal measure. The first pattern of widespread change was driven mainly by RD suggesting that the HD gene‐mutation may contribute to widespread demyelination (Fox et al., 2011), which given the correlation of this pattern with age in both controls and gene‐carriers could be an amplification of a pattern seen in normal ageing. This overall effect is reflected in the correlation between the PC score and both cognitive and motor performance. The difference in the strength of these associations echoes the accentuated effect of naturally‐occurring age‐related increased diffusivity in those with the HD gene. Increased AD in sensorimotor tracts, coupled with an apparent inverse relationship between RD and AD was similar to that identified previously (Orth et al., 2016). Although data were from the same cohort and therefore, some correspondence in findings is expected, this pattern is nevertheless consistent across both hemispheres of the sensorimotor network with earlyHD showing the highest levels of AD. Again, this was reflected in the strong correlations between increased AD, not RD, in the sensorimotor tracts and motor performance. Given that motor dysfunction is one of the key clinical presentations of HD, it would appear that AD increases are associated with motor deterioration seen in HD (Müller et al., 2016) and may reflect degeneration in the main fiber direction within motor pathways and subsequent disorganization leading to deficits in motor performance. In both cases however, we must proceed with caution given the considerable uncertainty associated with the biological interpretation of diffusivity changes (Jones et al., 2013). Taken together, our data suggest that HD pathogenesis increases RD across widespread networks, and AD in sensorimotor connections as part of a wider structural phenotype of earlyHD. Independent of key contributors to HD pathogenesis, RD increases in cognitive network‐based connections distinguished earlyHD from preHD or controls. This suggests that the HD mutation exerts effects on top of naturally‐occurring white matter tract microstructural variability. In addition, age‐related effects on cognitive tract microstructure independently modify HD pathogenesis, the identification of which may be valuable in understanding disease modification.

## AUTHOR CONTRIBUTIONS

Track‐On HD Investigators: A Coleman, J Decolongon, M Fan, T Koren (University of British Columbia, Vancouver); C Jauffret, D Justo, S Lehericy, K Nigaud, R Valabrègue (ICM and APHP, Pitié‐ Salpêtrière University Hospital, Paris); S Klöppel, E Scheller, L Minkova (University of Freiberg, Freiberg), A Schoonderbeek, E P ‘t Hart (Leiden University Medical Centre, Leiden); C Berna, M Desikan, R Ghosh, D Hensman Moss, E Johnson, P McColgan, G Owen, M Papoutsi, A Razi, J Read, (University College London, London); D Craufurd (Manchester University, Manchester); R Reilmann, N Weber (George Huntington Institute, Munster); H Johnson, J D Long, J Mills (University of Iowa, Iowa); J Stout, I Labuschagne (Monash University, Melbourne); G B Landwehrmeyer, I Mayer (Ulm University, Ulm).

## Supporting information

Additional Supporting Information may be found online in the supporting information tab for this article.

Supporting Information
