# Supplementary material for: Natural biological variation of white matter microstructure is accentuated in Huntington's disease
Source: Hum Brain Mapp. 2018 Apr 22;39(9):3516–27. doi: 10.1002/hbm.24191 (PMC6099203; doi:10.1002/hbm.24191)
Supplement: Supplementary file 1 — Supporting Information [file HBM-39-3516-s001.docx]

**Supplementary Information**

**Supplementary Tables**

*Supplementary Table 1*

| **Tract** | **Hemi** | **Region One** | **Toolbox** | **Region Two** | **Exclusion Masks** |
| --- | --- | --- | --- | --- | --- |
| Primary Motor Cortex to Motor Thalamus | R | Right M1 | AT | Right Motor Thalamus | Midbrain and LH |
|  | L | Left M1 | AT | Left Motor Thalamus | Midbrain and RH |
| Premotor Cortex to Motor Thalamus | R | Right PMC | AT | Right Motor Thalamus | Midbrain and LH |
|  | L | Left PMC | AT | Left Motor Thalamus | Midbrain and RH |
| Somatosensory Cortex to Somatosensory Thalamus | R | Right S1 | AT | Right Somatosensory Thalamus | Midbrain and LH |
|  | L | Left S1 | AT | Left Somatosensory Thalamus | Midbrain and RH |
| Primary Motor Cortex to Putamen | R | Right M1 | AT | Right Putamen | Midbrain, LH, Frontal Anterior Slice |
|  | L | Left M1 | AT | Left Putamen | Midbrain, RH, Frontal Anterior Slice |
| Premotor Cortex to Putamen | R | Right PMC | AT | Right Putamen | Midbrain, LH, Frontal Anterior Slice |
|  | L | Left PMC | AT | Left Putamen | Midbrain, RH, Frontal Anterior Slice |
| Somatosensory Cortex to Putamen | R | Right S1 | AT | Right Putamen | Midbrain, LH, Posterior Slice |
|  | L | Left S1 | AT | Left Putamen | Midbrain, RH, Posterior Slice |
| Primary Visual Cortex to Visual Thalamus | R | Right V1 | AT | Right Visual Thalamus | Midbrain, LH, Lateral Slice |
|  | L | Left V1 | AT | Left Visual Thalamus | Midbrain, RH, Lateral Slice |
| Posterior Parietal Cortex to Parietal Thalamus | R | Right PPC | WFU | Right Parietal Thalamus | Midbrain, LH, Thalamus, Superior Pos.Slice |
|  | L | Left PPC | WFU | Left Parietal Thalamus | Midbrain, RH, Thalamus, Superior Pos.Slice |
| Dorsolateral Prefrontal Cortex to Dorsolateral Prefrontal Thalamus | R | Right DLPFC | WFU | Right DLPF Thalamus | Midbrain, LH, Thalamus |
|  | L | Left DLPFC | WFU | Left DLPF Thalamus | Midbrain, RH, Thalamus |
| Dorsolateral Prefrontal Cortex to Caudate | R | Right DLPFC | WFU | Right Caudate | Midbrain, LH, Thalamus |
|  | L | Left DLPFC | WFU | Left Caudate | Midbrain, RH, Thalamus |
| Inferior Lateral Fasciculus | R | Right S1 | AT | Right Temporal Pole | LH |
|  | L | Left S1 | AT | Left Temporal Pole | RH |
| Inferior Fronto-Occipital Fasciculus | R | Right V1 | AT | Right External Capsule | LH |
|  | L | Left V1 | AT | Left External Capsule | RH |
| Uncinate Fasciculus | R | Right Temporal Pole | WFU | Right External Capsule | Medial Slice |
|  | L | Left Temporal Pole | WFU | Left External Capsule | Medial Slice |
| Mid Cingulum | R | Right Mid Cingulum | FSL |  | Midbrain and LH |
|  | L | Left Mid Cingulum | FSL |  | Midbrain and RH |
| Posterior Cingulum | R | Right Posterior Cingulum | FSL |  | Midbrain and LH |
|  | L | Left Posterior Cingulum | FSL |  | Midbrain and RH |
| Mid Corpus Callosum |  | Mid CC | FSL |  | Posterior Slice |
| Posterior Corpus Callosum |  | Posterior CC | FSL |  | Posterior Slice |

Tractography Information: Details of seed regions of interest, toolbox used to create the seed region and exclusion masks for each tract

*Abbreviations: M1: Primary Motor Cortex, S1: Primary Somatosensory Cortex, V1: Primary Visual Cortex, PMC: Premotor Cortex, PPC: Posterior Parietal Cortex, CC: Corpus Callosum, DLPFC: Dorsolateral Prefrontal Cortex. AT: Anatomy Toolbox, WFU: WFU PickAtlas*

*Supplementary Table 2*

Principal Component Analysis results for independent analysis on the controls and HD groups

|  | **CONTROLS** | | | **preHD+early HD** | | |
| --- | --- | --- | --- | --- | --- | --- |
|  | **Factor1** | **Factor2** | **Factor3** | **Factor1** | **Factor2** | **Factor3** |
| **FA_M1_ThalL** | . | 0.74313 | . | -0.44372 | 0.6527 | . |
| **FA_M1_ThalR** | . | 0.72925 | . | -0.49075 | 0.5471 | . |
| **FA_M1_PutL** | . | 0.80327 | . | -0.4226 | 0.6987 | . |
| **FA_M1_PutR** | . | 0.76542 | . | . | 0.7477 | . |
| **FA_PMC_ThalL** | . | 0.76398 | . | . | 0.7002 | . |
| **FA_PMC_ThalR** | . | 0.7847 | . | -0.46254 | 0.551 | . |
| **FA_PMC_PutL** | . | 0.83154 | . | -0.44607 | 0.6636 | . |
| **FA_PMC_PutR** | . | 0.76873 | . | -0.43426 | 0.6979 | . |
| **FA_S1_ThalL** | . | 0.74762 | . | -0.40375 | 0.6717 | . |
| **FA_S1_ThalR** | . | 0.65365 | -0.49924 | -0.42943 | 0.5736 | . |
| **FA_S1_PutL** | . | 0.7923 | . | -0.44813 | 0.6838 | . |
| **FA_S1_PutR** | . | 0.76428 | . | . | 0.7005 | . |
| **FA_mCC** | -0.53959 | 0.47746 | . | -0.77611 | . | . |
| **FA_pCC** | -0.64556 | . | . | -0.70519 | . | . |
| **FA_MidCingL** | -0.54639 | . | . | -0.59202 | . | . |
| **FA_MidCingR** | . | . | . | -0.61225 | . | . |
| **FA_PostCingL** | -0.49797 | . | . | -0.62898 | . | . |
| **FA_PostCingR** | -0.42609 | . | . | -0.60617 | . | . |
| **FA_DLCaudL** | . | 0.42699 | 0.45398 | -0.54487 | 0.4115 | . |
| **FA_DLCaudR** | . | 0.52069 | 0.60145 | -0.4567 | . | 0.60047 |
| **FA_DLThalL** | . | . | 0.64904 | . | . | 0.67441 |
| **FA_DLThalR** | . | 0.5033 | 0.66406 | . | . | 0.79467 |
| **FA_PPCThalL** | -0.73402 | . | . | -0.50148 | . | . |
| **FA_PPCThalR** | -0.65824 | . | . | -0.4598 | . | . |
| **FA_IFOFL** | -0.66964 | . | . | -0.70686 | . | . |
| **FA_IFOFR** | -0.55841 | . | . | -0.65822 | . | . |
| **FA_ILFL** | -0.68179 | . | . | -0.70421 | . | . |
| **FA_ILFR** | -0.62764 | . | . | -0.6528 | . | . |
| **FA_UFL** | -0.46808 | . | . | -0.47574 | . | . |
| **FA_UFR** | . | . | . | -0.48252 | . | . |
| **FA_V1ThalL** | -0.52198 | . | . | -0.61148 | . | . |
| **FA_V1ThalR** | -0.60097 | . | . | -0.47471 | . | . |
| **AD_M1_ThalL** | 0.58072 | 0.65715 | . | . | 0.7667 | . |
| **AD_M1_ThalR** | 0.5445 | 0.66134 | . | . | 0.7909 | . |
| **AD_M1_PutL** | 0.60913 | 0.55734 | . | 0.49075 | 0.6925 | . |
| **AD_M1_PutR** | 0.57242 | 0.66983 | . | 0.4656 | 0.7227 | . |
| **AD_PMC_ThalL** | 0.51054 | 0.64558 | . | . | 0.7902 | . |
| **AD_PMC_ThalR** | 0.58483 | 0.58949 | . | . | 0.7908 | . |
| **AD_PMC_PutL** | 0.69242 | 0.50137 | . | 0.48514 | 0.6825 | . |
| **AD_PMC_PutR** | 0.64129 | 0.49293 | . | 0.45251 | 0.6686 | . |
| **AD_S1_ThalL** | 0.56031 | 0.6397 | . | . | 0.7505 | . |
| **AD_S1_ThalR** | 0.56959 | 0.64145 | . | . | 0.794 | . |
| **AD_S1_PutL** | 0.63412 | 0.52221 | . | 0.46387 | 0.7207 | . |
| **AD_S1_PutR** | 0.62658 | 0.61964 | . | 0.53491 | 0.6469 | . |
| **AD_mCC** | 0.5517 | 0.46409 | . | 0.56761 | 0.4309 | . |
| **AD_pCC** | 0.55892 | . | . | 0.59199 | . | . |
| **AD_MidCingL** | 0.60014 | . | . | . | 0.4927 | 0.51147 |
| **AD_MidCingR** | 0.58995 | 0.42159 | . | 0.51474 | 0.4282 | 0.51979 |
| **AD_PostCingL** | 0.4666 | . | . | . | . | 0.62382 |
| **AD_PostCingR** | 0.47876 | 0.50209 | . | . | . | 0.54354 |
| **AD_DLCaudL** | 0.65506 | . | . | 0.63586 | . | . |
| **AD_DLCaudR** | 0.60746 | . | . | 0.66483 | . | . |
| **AD_DLThalL** | 0.66211 | . | . | 0.65165 | 0.4479 | . |
| **AD_DLThalR** | 0.59126 | . | . | 0.66054 | . | . |
| **AD_PPCThalL** | 0.60374 | . | . | 0.63797 | . | . |
| **AD_PPCThalR** | 0.53932 | . | . | 0.51132 | 0.4654 | . |
| **AD_IFOFL** | 0.46213 | . | . | 0.55076 | . | . |
| **AD_IFOFR** | . | . | . | 0.55004 | . | . |
| **AD_ILFL** | 0.43006 | . | . | 0.50389 | . | . |
| **AD_ILFR** | . | . | . | 0.52176 | 0.4228 | . |
| **AD_UFL** | 0.43566 | . | . | . | . | . |
| **AD_UFR** | . | . | . | 0.62665 | . | . |
| **AD_V1ThalL** | . | . | . | 0.45038 | . | . |
| **AD_V1ThalR** | . | 0.49324 | . | . | 0.4299 | . |
| **RD_M1_ThalL** | 0.68716 | . | . | 0.70359 | . | . |
| **RD_M1_ThalR** | 0.57137 | . | . | 0.70319 | . | . |
| **RD_M1_PutL** | 0.74971 | -0.45286 | . | 0.78674 | . | . |
| **RD_M1_PutR** | 0.6542 | . | . | 0.69866 | . | . |
| **RD_PMC_ThalL** | 0.55656 | -0.43681 | . | 0.63261 | . | . |
| **RD_PMC_ThalR** | 0.47916 | -0.55744 | . | 0.727 | . | . |
| **RD_PMC_PutL** | 0.69996 | -0.53329 | . | 0.78552 | . | . |
| **RD_PMC_PutR** | 0.64373 | -0.4834 | . | 0.74001 | . | . |
| **RD_S1_ThalL** | 0.71552 | . | . | 0.72939 | . | . |
| **RD_S1_ThalR** | 0.64211 | . | 0.4659 | 0.63517 | . | 0.49433 |
| **RD_S1_PutL** | 0.76885 | . | . | 0.79435 | . | . |
| **RD_S1_PutR** | 0.75839 | . | . | 0.78251 | . | . |
| **RD_mCC** | 0.80224 | . | . | 0.85441 | . | . |
| **RD_pCC** | 0.80854 | . | . | 0.8167 | . | . |
| **RD_MidCingL** | 0.75911 | . | . | 0.64526 | . | . |
| **RD_MidCingR** | 0.73789 | . | . | 0.77639 | . | . |
| **RD_PostCingL** | 0.78051 | . | . | 0.75687 | . | . |
| **RD_PostCingR** | 0.72808 | . | . | 0.79899 | . | . |
| **RD_DLCaudL** | 0.62901 | . | -0.44694 | 0.72442 | . | . |
| **RD_DLCaudR** | 0.62685 | . | . | 0.75623 | . | -0.42307 |
| **RD_DLThalL** | . | . | -0.61767 | . | . | -0.66889 |
| **RD_DLThalR** | . | -0.42065 | -0.65034 | . | . | -0.75914 |
| **RD_PPCThalL** | 0.88633 | . | . | 0.81964 | . | . |
| **RD_PPCThalR** | 0.78622 | . | . | 0.72144 | . | 0.43653 |
| **RD_IFOFL** | 0.83072 | . | . | 0.85611 | . | . |
| **RD_IFOFR** | 0.75489 | . | . | 0.86629 | . | . |
| **RD_ILFL** | 0.79668 | . | . | 0.76664 | . | . |
| **RD_ILFR** | 0.75516 | . | . | 0.83127 | . | . |
| **RD_UFL** | 0.6255 | . | . | 0.60946 | . | . |
| **RD_UFR** | . | . | . | 0.62068 | . | . |
| **RD_V1ThalL** | 0.71456 | . | . | 0.76697 | . | . |
| **RD_V1ThalR** | 0.66647 | . | . | 0.64907 | . | . |

*Abbreviations: M1: Primary Motor Cortex, S1: Primary Somatosensory Cortex, V1: Primary Visual Cortex, PMC: Premotor Cortex, PPC: Posterior Parietal Cortex, CC: Corpus Callosum, DLPFC: Dorsolateral Prefrontal Cortex, IFOF: Inferior Fronto-Occipital Gyrus, ILF: Inferior Frontal Gyrus, UF: Uncinate Fasciculus. FA: Fractional Anisotropy, MD: Mean Diffusivity, RD: Radial Diffusivity, AD: Axial Diffusivity.*
